# Prospective evaluation of the G8 screening tool for prognostication of survival in elderly patients with lung cancer: A single-institution study

**DOI:** 10.1371/journal.pone.0210499

**Published:** 2019-01-17

**Authors:** Yoko Agemi, Tsuneo Shimokawa, Jiichiro Sasaki, Kazuhito Miyazaki, Yuki Misumi, Akira Sato, Shinzi Aida, Mari Ishii, Yukiko Nakamura, Katsuhiko Naoki, Hiroaki Okamoto

**Affiliations:** 1 Department of Respiratory Medicine, Yokohama Municipal Citizen’s Hospital, Yokohama, Kanagawa, Japan; 2 Department of Respiratory Medicine, Kitasato University School of Medicine, Sagamihara, Kanagawa, Japan; 3 Department of Cancer Strategy, Cancer Control Center, Osaka International Cancer Institute, Osaka, Japan; 4 Department of Medical Oncology, Yokohama Municipal Citizen’s Hospital, Yokohama, Kanagawa, Japan; University of South Alabama Mitchell Cancer Institute, UNITED STATES

## Abstract

The G8 questionnaire is a quick and easy-to-use screening tool. Several studies reported that the G8 questionnaire had a high sensitivity for predicting abnormalities in the full comprehensive geriatric assessment and predicted functional decline and survival in elderly cancer patients. The present study aimed to evaluate the role of the G8 questionnaire for predicting clinical outcomes and overall survival (OS) in elderly patients with lung cancer, who received chemotherapy or chemoradiotherapy. The data of 101 lung cancer patients aged ≥70 years, who were hospitalized between September 2011 and August 2014, were analyzed. Of these patients (median age, 77 years), 83 (82%) had impaired G8 scores. The proportion of patients with an impaired G8 score was significantly higher in patients aged ≥80 years than those aged <80 years (p = 0.04). All 18 patients with a normal G8 score possessed an Eastern Cooperative Oncology Group performance status (ECOG PS) of 0 or 1, and none of the patients with a normal G8 score had an ECOG PS of ≥2 (p < 0.0001). An impaired G8 score tended to correlate with a relative dose intensity of <0.65 in patients who received chemotherapy or chemoradiotherapy (p = 0.05, odds ratio = 5.40). In the univariate analysis, an ECOG PS of ≥2 and an impaired G8 score were significantly associated with a poor OS (p = 0.009 and p = 0.003, respectively). Moreover, in the multivariate analysis, an ECOG PS of ≥2 (HR 2.55; 95% CI, 1.23–5.30; p = 0.01) and an impaired G8 score (HR 3.86; 95% CI, 1.44–13.36; p = 0.006) were remained independent prognostic factor for OS. G8 screening tool is useful for the prognostication of elderly lung cancer patients treated with chemotherapy. These finding suggest that the G8 questionnaire could be a useful tool in treatment decision-making to predict prognosis and prevent patients from receiving inappropriate anti-cancer treatment near the end of life.

## Introduction

Alongside the aging of the global population, the number of elderly persons with lung cancer has been increasing. Recent data of the Surveillance, Epidemiology, and End Results program from the United States showed that patients aged ≥70 years accounted for 47% of all lung cancer cases [1]. This trend is similar to Japan, where more than 60% of new lung cancer cases are seen in individuals aged ≥65 years [2]. Furthermore, more than 75% of deaths from lung cancer in Japan are occurring in persons aged ≥65 years [3]. While the number of elderly patients with lung cancer is increasing, treatment guidelines are based on clinical trials conducted in healthy elderly participants. Therefore, these guidelines are difficult to apply to general clinical settings in which elderly, fragile, and heterogeneous populations are treated [4].

The Comprehensive Geriatric Assessment (CGA) is a widely used method to determine the medical, psychological, and functional capabilities of older patients and different components of CGA can be useful to predict toxicity [4, 5] and functional decline [6]. The International Society of Geriatric Oncology recommends that a CGA should be used in elderly cancer patients to select fit elderly patients who are able to receive standard treatment [7]. However, the CGA is time consuming [8, 9]. In contrast, the G8 questionnaire is a quick and easy-to-use screening tool that takes less than 5 minutes to administer. The G8 screening tool consists of 7 items from the mini nutritional assessment questionnaire and age (S1 Table) [10]. Several recent studies showed that the G8 questionnaire had a high sensitivity for predicting abnormalities in the full CGA [11]. Screening tools including G8 do not replace CGA but are recommended in a busy practice in order to identify those patients in need of full CGA [12]. Besides being a convenient screening tool for geriatric assessment, the G8 questionnaire has also been claimed to be a valuable tool for predicting survival. Several studies reported that the G8 detected functional decline and predicted survival in elderly cancer patients [13, 14]. Regarding lung cancer, a retrospective study conducted with 142 elderly lung cancer patients demonstrated that potentially frail patients, identified by an impaired G8 or Identification of Seniors at Risk for Hospitalized Patients (ISAR-HP), and higher disease stage had a significantly greater risk of 1-year mortality in the Cox regression analysis. Furthermore, analyzing the screening instruments separately showed that the G8 had an independent relation with 1-year mortality and the ISAR-HP did not [15]. Although the G8 tool has been extensively investigated in several elder cancers including solid and liquid tumors, its prediction ability in lung cancers is still elusive.

The present study aimed to validate the role of the G8 questionnaire for predicting overall survival (OS) in elderly lung cancer patients, who received chemotherapy (CT). In addition, we aimed to evaluate the association between G8 scores and predictive factors for unfavorable clinical outcomes, such as severe adverse events (SAEs), cessation of treatment (COT), and relative dose intensity (RDI) <0.65.

## Patients and methods

### Study design and population

In this prospective cohort study, we enrolled 101 lung cancer patients aged ≥70 years who were candidates for CT, radiotherapy (RT), or chemoradiotherapy (CRT) and were hospitalized at Yokohama Municipal Citizen’s Hospital between September 2011 and August 2014. Since only hospitalized patients were included in this study, patients who were prescribed oral anti-cancer agents and initiated treatment in an outpatient setting were excluded. Furthermore, patients that physicians considered not suitable for receiving a G8 questionnaire were also excluded. This study was approved by the ethics committee of Yokohama Municipal Citizen’s Hospital (18-04-03). Participants provided their verbal informed consent to participate in this study. Because G8 examination was neither invasive nor interventional, our ethics committee recommended not to get written informed consent in this observational study.

### G8 screening and other measures

All participants underwent G8 screening by physicians before the beginning of treatment (S1 Table). The maximum score of the G8 is 17 points, and a score of ≤14 is defined as an impaired G8 score, according to previous studies that analyzed the association between the G8 score and OS [10, 16].

In addition to the G8 scores, we collected the following patient characteristics: age, sex, histology, Eastern Cooperative Oncology Group performance status (ECOG PS) and stage. Furthermore, we investigated the associations between G8 scores and SAEs, RDI, COT, and OS in patients who received CT or CRT. A score of SAEs were defined as Grade 3–5 non-hematologic and Grade 4–5 hematologic adverse events. RDI was defined as the ratio of received to expected chemotherapy doses, and RDI was evaluated for initial 2 months of therapy. We set cut-off value of RDI to 0.65, because a recent study that reported a relationship between components of the CGA, chemotherapy dose intensity, and OS in colorectal cancer set cut-off value of RDI to 0.65 and 0.85 [17]. OS was defined as the period from the date of the diagnosis of lung cancer to the date of death from any cause or the last follow-up. The treatment strategy was decided based on the patient’s ECOG PS, age, and status of organ function, regardless of the G8 score.

### Statistical analysis

The association between G8 score and patient characteristics was analyzed by the Fisher’s exact test. Determination of predictive factors for unfavorable clinical outcomes such as SAE, COT, and RDI <0.65 were performed using logistic regression analysis. Cumulative survival rates were calculated by the Kaplan-Meier method. The log-rank test was used to evaluate survival difference between the groups (normal G8 vs. impaired G8). Univariate analysis and multivariate analysis were performed using Cox regression analysis to evaluate the prognostic value of six clinically selected variable [G8 score, age (<80 vs. ≥80), sex, histology (NSCLC vs. SCLC), disease stage (I-III vs. IV)) and ECOG PS (0–1 vs. ≥2)]. The analyses of clinical outcomes were performed only in patients receiving CT or CRT. All data were analyzed with the JMP 9 software (SAS Institute Inc., Cary, NC, USA). Differences were considered significant when the p value was less than 0.05.

## Results

### Patient characteristics

Patient characteristics are listed in Table 1. The median age of the patients was 79 years-old (range, 70–95 years-old), with 19.8% being women. Moreover, there were 45 (44.6%) and 56 (55.4%) patients aged ≥80 years and <80 years, respectively. Adenocarcinoma was the most common histologic type, accounting for 45.6% of the patients, followed by squamous cell carcinoma (29.7%), and small cell carcinoma (18.8%). The ECOG PS score was 0, 1, 2, and 3 in 8 (7.9%), 50 (49.5%), 33 (32.7%), and 10 (9.9%) patients, respectively. At the time of evaluation, 85 patients had newly diagnosed lung cancer; of these, 38 patients had stage I–III disease and 47 patients had stage IV disease. 16 patients had recurrent disease; of these, 2 patients had stage I–III disease and 14 patients had stage IV disease. An impaired G8 score was found in 82.2% of all patients. The most common treatment was CT (58.4% of the patients), followed by RT (24.8%), best supportive care (BSC) only (9.9%), and CRT (6.9%).

**Table 1:** Patient characteristics (n = 101).

Clinical Characteristic		No. of Patients (n = 101)	%
Age	Median age (range)	79 (70–95)	
Sex	Male	81	80.2
	Female	20	19.8
Histology	Adenocarcinoma	46	45.6
	Squamous cell carcinoma	30	29.7
	Large cell carcinoma	1	1.0
	NOS	5	4.9
	SCLC	19	18.8
ECOG PS	0	8	7.9
	1	50	49.5
	2	33	32.7
	3	10	9.9
Stage	I	5	5.0
	II	7	6.9
	III	26	25.7
	IV	47	46.5
	Recurrence	16	15.9
G8 screening score	Median (range)	12 (2–17)	
	≤14	83	82.2
Treatment Received	RT	25	24.8
	CT	69	58.4
	CRT	7	6.9
	BSC	10	9.9

NOS, not otherwise specified; SCLC, small cell lung cancer; ECOG, Eastern Cooperative Oncology Group; PS, Performance status; RT, radiotherapy; CT, chemotherapy; CRT, chemoradiotherapy; BSC, best supportive care.

### Association between G8 scores and patient characteristics

The associations between G8 score and patient characteristics are shown in Table 2. The proportion of patients with an impaired G8 score was significantly higher in patients aged ≥80 years than those aged <80 years (p = 0.04). All 18 patients with a normal G8 score possessed an ECOG PS of 0 or 1, and none of the patients with a normal G8 score had an ECOG PS of ≥2 (p < 0.0001). We observed no differences in sex, histology, stage, and the presence or absence of CT between patients with a normal and an impaired G8 score. Of all the analyzed patients, 76 (75%) received CT or CRT. The associations between G8 scores and those 76 patient characteristics are listed in Table 3. As with the analysis of the entire cohort, all 18 patients with a normal G8 score possessed an ECOG PS of 0 or 1, and none of the patients with a normal G8 score had an ECOG PS of ≥2 (p < 0.0001)

**Table 2:** G8 scores and patient characteristics (n = 101).

		Total	Normal G8 scores	Impaired G8 scores	*p* value
		n = 101	n = 18	n = 83	
Age	80>	56	14 (25%)	42 (75%)	0.04*
	≥80	45	4 (9%)	41 (91%)	
Sex	Male	81	17 (21%)	64 (79%)	0.11
	Female	20	1 (5%)	19 (95%)	
Histology	NSCLC	82	14 (17%)	68 (83%)	0.74
	SCLC	19	4 (21%)	15 (79%)	
ECOG PS	0–1	58	18 (31%)	40 (69%)	<0.0001*
	≥2	43	0 (0%)	43 (100%)	
Stage	I–III	40	6 (15%)	34 (85%)	0.61
	IV	61	12 (20%)	49 (80%)	
Treatment received	Non-chemotherapy	25	0 (0%)	25 (100%)	0.11
	Chemotherapy	76	18 (24%)	58 (76%)	

NSCLC, non-small cell lung cancer; SCLC, small cell lung cancer; ECOG, Eastern Cooperative Oncology Group; PS, Performance status.

*Statistically significant (p < 0.05)

**Table 3:** G8 scores and patient characteristics in the patients treated with chemotherapy (n = 76).

		Total	Normal G8 scores	Impaired G8 scores	*p* value
		n = 76	n = 18	n = 58	
Age	80>	48	14 (29%)	34 (71%)	0.13
	≥80	28	4 (14%)	24 (86%)	
Sex	Male	63	17 (27%)	46 (73%)	0.10
	Female	13	1 (8%)	12 (92%)	
Histology	NSCLC	58	14 (24%)	44 (76%)	0.86
	SCLC	18	4 (22%)	14 (78%)	
ECOG PS	0–1	52	18 (35%)	34 (65%)	<0.0001*
	≥2	24	0 (0%)	24 (100%)	
Stage	I–III	23	6 (26%)	17 (74%)	0.74
	IV	53	12 (23%)	41 (77%)	

NSCLC, non-small cell lung cancer; SCLC, small cell lung cancer; ECOG, Eastern Cooperative Oncology Group; PS, Performance status.

*Statistically significant (p < 0.05)

### Prognostic value of the G8 and other clinical parameters

For those patients who received CT or CRT, we evaluated the predictive factors for clinical outcomes including SAE, COT caused by SAEs, and an RDI <0.65 (Table 4). Logistic regression analysis revealed that stage (I–III vs. IV) and an impaired G8 tended to be correlated with an RDI <0.65 (p = 0.09, OR = 3.41, 95% confidence interval [CI]: 0.84–23.14; p = 0.05, OR = 5.40, 95% CI: 0.97–101.76, respectively).

**Table 4:** Prognostic value of G8 and other patient characteristics in the patients treated with chemotherapy (n = 76).

	Cut-off	SAE OR (95% CI)	*P*	COT because of AE OR (95% CI)	*P*	RDI <0.65 OR (95% CI)	*P*
Age	≥80 vs. <80	0.84 (0.32–2.25)	0.73	1.52 (0.51–4.47)	0.45	1.67 (0.52–5.28)	0.38
Sex	Male vs. Female	0.72 (0.18–2.49)	0.61	1.87 (0.44–12.94)	0.42	1.43 (0.33–9.98)	0.66
Histology	SCLC vs. NSCLC	1.70 (0.56–5.91)	0.35	0.32 (0.04–1.33)	0.13	0.43 (0.06–1.80)	0.27
ECOG PS	2–3 vs. 0–1	0.83 (0.29–2.44)	0.73	2.20 (0.69–6.82)	0.18	2.24 (0.98–7.36)	0.19
Stage	IV vs. I–III	1.50 (0.54–4.08)	0.43	2.63 (0.76–12.32)	0.13	3.41 (0.84–23.14)	0.09
G8 Score	≤14 vs. >14	0.81 (0.25–2.43)	0.72	3.05 (0.75–20.65)	0.13	5.40 (0.97–101.76)	0.05

SAE, severe adverse event; COT, cessation of treatment; AE, adverse event; RDI, relative dose intensity; OR, odds ratio; CI, confidence interval; NSCLC, non-small cell lung cancer; SCLC, small cell lung cancer; ECOG, Eastern Cooperative Oncology Group; PS, Performance status.

**Fig 1:**
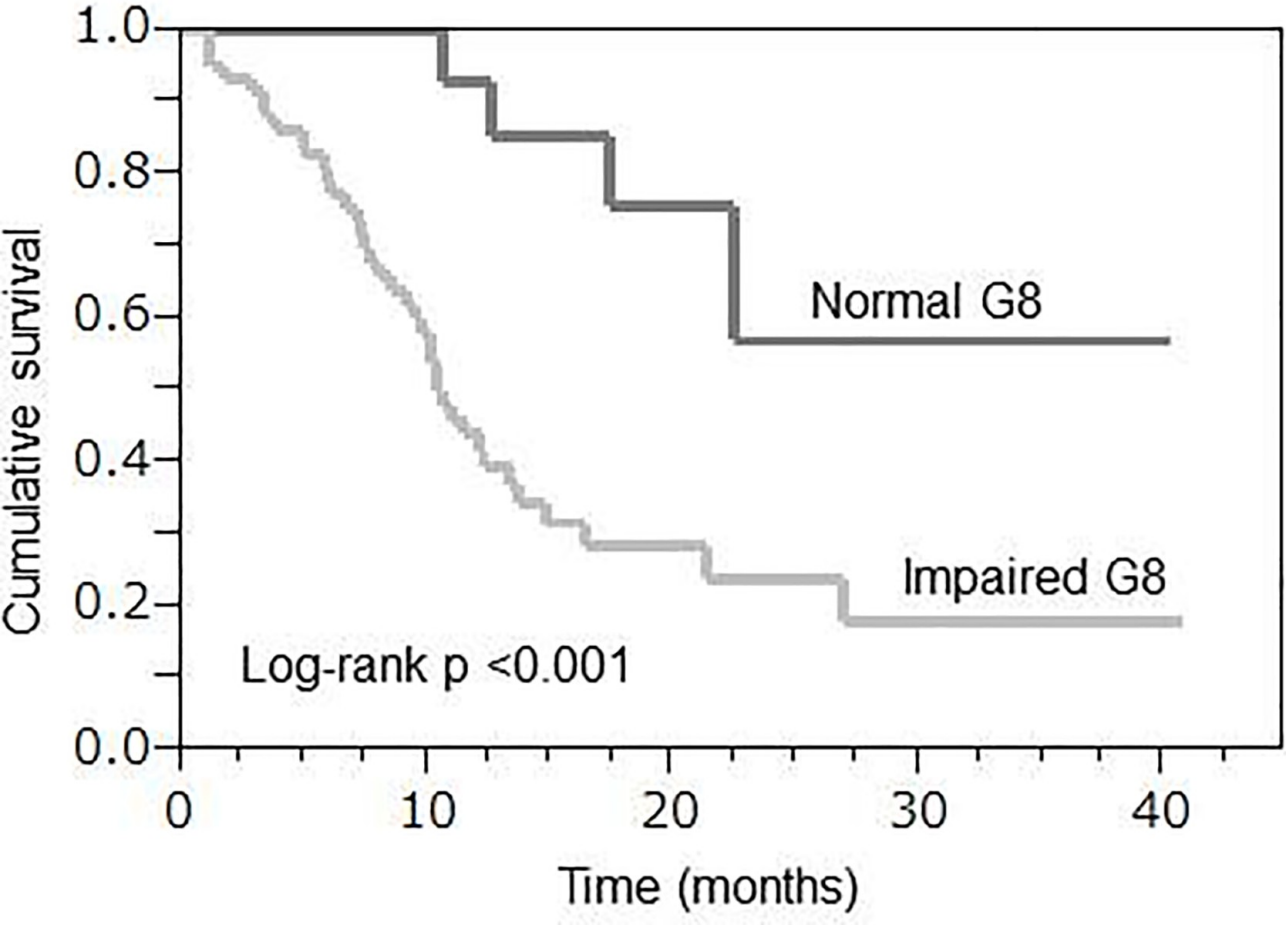
Overall survival according to the G8 score in patients who received chemotherapy (n = 76). Kaplan-Meier analyses for overall survival according to the G8 score.

For evaluating OS, the median follow-up time was 12.8 months (range, 1.1–40.8 months). We compared OS between patients with normal and impaired G8 scores and found that it showed significant prognostic value (log-rank p <0.001, Fig 1). The median survival time was 10.6 months for patients with an impaired G8 score, whereas a median survival time was not achieved for patients with a normal G8 score. In the univariate analysis, ECOG PS of ≥2 (HR 2.54; 95% CI, 1.28–4.88; p = 0.009) and an impaired G8 score (HR 4.87; 95% CI, 1.94–16.32; p = 0.003) were significantly associated with a poor OS. In the multivariate analysis, ECOG PS of ≥2 (HR 2.55; 95% CI, 1.23–5.30; p = 0.01) and an impaired G8 score (HR 3.86; 95% CI, 1.44–13.36; p = 0.006) were remained independent prognostic factor for OS (Table 5).

**Table 5:** Univariate and multivariate survival analyses in patients who received chemotherapy (n = 76).

		Univariate analysis		Multivariate analysis	
	Cut-off	OS HR (95% CI)	*p*	OS HR (95% CI)	*p*
Age	≥80 vs. <80	0.93 (0.48–1.73)	0.82	0.90 (0.46–1.69)	0.74
Sex	Male vs. Female	0.73 (0.35–1.71)	0.45	1.10 (0.38–1.93)	0.81
Histology	SCLC vs. NSCLC	0.83 (0.37–1.67)	0.62	0.53 (0.23–1.15)	0.11
ECOG PS	2–3 vs. 0–1	2.54 (1.28–4.88)	0.009*	2.55 (1.23–5.30)	0.01*
Stage	IV vs. I–III	1.61 (0.80–3.59)	0.19	1.39 (0.68–3.14)	0.38
G8 Score	≤14 vs. >14	4.87 (1.94–16.32)	0.003*	3.86 (1.44–13.36)	0.006*

OS, overall survival; HR, hazard ratio; CI, confidence interval; NSCLC, non-small cell lung cancer; SCLC, small cell lung cancer; ECOG, Eastern Cooperative Oncology Group; PS, Performance status.

*Statistically significant (p < 0.05)

## Discussion

This prospective cohort study was designed to evaluate the role of the G8 score in predicting clinical outcomes and OS in elderly patients with lung cancer. Moreover, this study was the first study to evaluate the association between G8 score and the predictive factors for unfavorable clinical outcomes such as SAE, COT, and RDI <0.65. As a result, we found that an impaired G8 score tended to correlate with an RDI <0.65. Moreover, our study revealed that the G8 score and ECOG PS were independent prognostic factors for OS.

Compared to the general population of elderly lung cancer patients in Japan, there are slightly more male than female in the male-to-female ratio observed in our study cohort. Generally, female patients have a high proportion of EGFR-positive lung cancer. Patients with EGFR-positive lung cancer commonly begin oral treatment as outpatients. These groups of patients are excluded from this study, since we examined hospitalized patients. Therefore, this is why there are slightly more male than female in this study. The histology of the patients in this study is representative of the general population of elderly lung cancer patients in Japan. In our study, 82.2% of the patients had impaired G8 scores; this is slightly higher or comparable to previous studies [10, 13, 18–20]. The results of the present study showed that the proportion of patients with an impaired G8 score was significantly higher in patients aged ≥80 years than those aged <80 years (p = 0.04). All 18 patients with a normal G8 score possessed an ECOG PS of 0 or 1, and none of the patients with a normal G8 score had an ECOG PS of ≥2 (p < 0.0001). When patients who received CT or CRT were analyzed, it was also shown that G8 score and ECOG PS score were significantly correlated to each other. This correlation between G8 score and ECOG PS score has been demonstrated by other studies [14, 21].

The G8 score was not found to be useful for predicting SAEs or COT, while an impaired G8 score tended to correlate with an RDI <0.65. We speculate the reason for this is as follows. When physicians determine the appropriate dosing for cytotoxic anticancer agents, they make decisions based not only on age and ECOG PS, but also functional decline. Age and ECOG PS alone may be insufficient to predict chemotherapy tolerance and clinical outcomes. Functional decline may be more important and it is associated with the G8 score in elderly patients and the reasons are as follows. The G8 screening tool puts weight on nutritional status and consists of mobility, neuropsychological problems, drugs prescribed, self-assessment health condition, and age, from the Mini-Nutritional Assessment questionnaire [22, 23]. We think that these items may be important factors for reflecting functional decline and predicting chemotherapy tolerance in elderly cancer patients. In order to prove this assumption is true, it is necessary to continue further research and increase the number of cases.

In the multivariate analysis, an ECOG PS score ≥2 and an impaired G8 score were remained independent prognostic factor for OS and an impaired G8 score (HR 2.55; 95% CI, 1.23–5.30; p = 0.01) was more strongly correlated with prognosis than PS score ≥2 (HR 3.86; 95% CI, 1.44–13.36; p = 0.006). It is reported that PS [24], weight loss [25], nutritional status [26], and inflammatory biomarkers [27] can predict survival in advanced lung cancer patients. As mentioned above, the G8 screening tool consists of assessments for food intake, weight loss, mobility, neuropsychological problems, drugs prescribed, self-assessment health condition, and age, from the Mini-Nutritional Assessment questionnaire [22, 23]. We speculate that evaluating patients using multiple aspects is reason why G8 was found to be a stronger prognostic factor than ECOG PS.

Each patient, and their associated cancer, has unique characteristics, among which lung cancer generally has a rapid disease course and poor overall survival [27, 28]. Therefore, it is important to specifically investigate each patient with lung cancer. Previously, Schulkes et al. found that potentially frail patients, identified by an impaired G8 or ISAR-HP, and higher disease stage had a significantly greater risk of 1-year mortality in the Cox regression analysis. Furthermore, analyzing the screening instruments separately showed that the G8 had an independent relation with 1-year mortality and the ISAR-HP did not [15]. The findings of our study are similar to this work, which supports G8 score as a prognostic factor of elderly lung cancer. However, our multivariate analysis did not include ISAR-HP score. In addition, several variables included into multivariate analysis were different from that of their studies. Nevertheless, similar results indicating that G8 was an independent prognostic factor in elderly lung cancer patients were obtained in both studies. Therefore, we believe that our results can add to the current knowledge.

Corre et al. reported a multicenter, open-label, phase III trial in 474 elderly patients aged ≥70 years with an ECOG PS score 0–2 and stage IV non-small cell lung cancer [29]. In this trial, patients were randomly assigned between chemotherapy allocation on the basis of PS and age (standard arm) and treatment allocation including chemotherapy and BSC on the basis of CGA (CGA arm). This study showed that treatment allocation on the basis of CGA failed to improve treatment failure free survival and OS, but slightly reduced treatment-related toxicity, although the CGA arm contained 23% of patients receiving BSC. Moreover, patients in the CGA arm, when compared to those in the standard arm, showed a significantly lower rate of all-grade toxicity (85.6% *vs*. 93.4%, p = 0.015) and a significantly lower rate of treatment failure resulting from toxicity (4.8% *vs*. 11.8%, p = 0.007). The fact that OS in the CGA arm was similar to that of the standard arm and the treatment-related toxicity was low despite the CGA arm containing 23% of patients receiving BSC alone, might indicate that frail patients in the CGA arm could avoid ineffective chemotherapy. What is revealed in this study is the possibility of using CGA to identify patients who should receive BSC without reducing their survival. However, since the CGA is time-consuming, economically low in rewards for most medical systems, and not necessary for all patients [30], applying this tool is burdensome and difficult to use for clinicians in their daily clinical practice. Therefore, it is important to examine whether the G8 questionnaire can be used as an alternative to the CGA to select patients who should receive BSC.

Our study has some limitations. First, our study included biases because the characteristics of the patients such as stage, histological type, and treatment received were heterogeneous. However, previous studies that reported the G8 questionnaire was a valuable predictive tool of survival also included patients with heterogeneous stages, treatments, and various types of solid cancers [19], various types of blood cancers [13], and both [14, 18]. Although bias cannot be excluded, it is interesting that the G8 score was a stronger prognostic factor than the ECOG PS; moreover, we were able to show that the prognostic value of the G8 remained in this heterogeneous population, which is a major strength of this study. Second, this study included a relatively low number of patients. Since this was an observational study, there was no sample size determination.

In conclusion, our prospective study found that an impaired G8 score tended to correlate with a RDI <0.65 in elderly lung cancer patients treated with chemotherapy. Moreover, we revealed that the G8 score was an independent prognostic factor for OS like ECOG PS. These finding suggest that the G8 questionnaire could be a useful tool in treatment decision-making to predict prognosis and prevent patients from receiving inappropriate anti-cancer treatment near the end of life. As this study was performed at a single institution and the number of cases was small, multi-center large-scale trials are necessary to confirm these results in elderly patients with lung cancer.

## Supporting information

S1 Table The G8 questionnaire. BMI, body mass index.
(DOCX)
