# Supplementary material for: Prospective evaluation of the G8 screening tool for prognostication of survival in elderly patients with lung cancer: A single-institution study
Source: PLoS One. 2019 Jan 17;14(1):e0210499. doi: 10.1371/journal.pone.0210499 (PMC6336333; doi:10.1371/journal.pone.0210499)
Supplement: S1 Table — BMI, body mass index. (DOCX) [file pone.0210499.s001.docx]

| **Items** | **Possible responses (score)** |
| --- | --- |
| 1. Has food intake declined over the past 3 months due to loss of appetite, digestive problems,　chewing, or swallowing difficulties? | 0＝severe decrease in food intake  1 = moderate decrease in food intake  2 = normal food intake |
| 1. Weight loss during the last 3 months? | 0 = weight loss > 3kg  1 = does not know  2 = weight loss between 1 and 3 kg  3 = no weight loss |
| 1. Mobility? | 0 = bed or chair bound  1 = able to get out of bed/chair but does not go out  2 = goes out |
| 1. Neuropsychological problem? | 0 = severe dementia or depression  1 = mild dementia or depression  2 = no psychological problems |
| 1. BMI？ (weight in kg)/(height in m^2^) | 0 = BMI ＜ 19  1 = 19 ≤ to < 21  2 = 21 ≦ to < 23  3 = BMI ≧ 23 |
| 1. Takes more than three prescription drugs per day? | 0 = yes  1 = no |
| 7 In comparison with other people of the same age, how  does the patient consider his/her health status? | 0 = not as good  0.5 = does not know  1 = as good   1. = better |
| 8 Age? | 0 = ＞85 years  1 = 80-85 years  2 = <80 years |
| Total score 0-17 | cutoff ≤ 14 |
